# Thermal Cameras for Overnight Measuring of Respiration in a Clinical Setting

**DOI:** 10.3390/s25195956

**Published:** 2025-09-24

**Authors:** Raquel Alves, Fokke van Meulen, Sebastiaan Overeem, Hennie Janssen, Pauline van Hirtum, Svitlana Zinger, Sander Stuijk

**Affiliations:** 1 Department of Electrical Engineering, Eindhoven University of Technology, 5600MB Eindhoven, The Netherlands; 2 Centre for Sleep Medicine Kempenhaeghe, 5590AB Heeze, The Netherlands

**Keywords:** thermal cameras, sleep, polysomnography, respiration rate, remote thermography

## Abstract

Thermal imaging is a non-contact method for monitoring respiration activity during sleep. In this study, we evaluated its clinical application during overnight recordings in a sleep clinic. Five thermal cameras were used to detect breaths, the estimated respiration rate (RR), and inter-breath intervals (IBIs) in seven adults undergoing diagnostic polysomnography (PSG). Forty-five minutes of recordings were selected, consisting of 12 motionless and event-free segments. The thermal videos were processed using an adapted pre-existing thermal video processing algorithm. The respiration signals generated with the thermal cameras were validated against simultaneously recorded signals from the PSG system, the current gold standard for monitoring sleep. The results show a mean absolute error (MAE) ranging between 0.64 and 0.91 breaths per minute for the RR. Breath detection showed a sensitivity of 96.3%, and a precision of 94.1%. The MAE obtained between IBIs was 0.48 s, and the mean IBI variability difference recorded was 3.9 percentage points. In addition, the results from this clinical study show that the use of all five cameras and a single camera revealed no statistically significant differences, demonstrating the work towards a robust system. This first study of thermal cameras for the assessment of respiration in a clinical setting shows us the potential application of thermal imaging in clinical practice for respiration monitoring and establishes a foundation for further implementation in assessing sleep-disordered breathing.

## 1. Introduction

Changes in respiration patterns, such as abnormal respiration rates, patterns, or reduced inhaled air volume, are often early indicators of serious medical conditions, including sleep disorders and respiratory failure [1,2,3]. Continuous and accurate monitoring of respiration during sleep is essential for the diagnosis and management of disorders such as obstructive sleep apnea and other sleep-related breathing disorders [4,5]. Non-contact methods for respiration monitoring are becoming increasingly valuable, offering the potential to improve patient comfort while ensuring reliable clinical assessments [6].

Thermography has gained increasing attention as a promising, contactless technique for respiration monitoring, offering several advantages over conventional methods [7]. Thermal cameras detect the infrared radiation naturally emitted by the human body. This allows for the visualization of temperature changes around the nose and mouth associated with inhalation and exhalation, and the temperature changes associated with respiration movements of the shoulders, thorax, and abdomen. Unlike traditional sensors for respiration monitoring, thermal imaging does not require physical contact with the patient, reducing discomfort, minimizing the risk of skin irritation, and preserving natural sleep behaviors. Moreover, thermal cameras can operate in complete darkness without the need for additional lighting, making them particularly suitable for overnight monitoring in clinical environments [6,7].

Nevertheless, despite its technical potential, the clinical application of thermal imaging remains limited. Most existing studies have focused on healthy individuals under controlled conditions, and only a few have used thermal systems in real clinical settings [7]. Pereira et al. [8,9,10] performed several studies where they demonstrated the viability of thermal cameras for respiration monitoring in healthy adults. However, these studies were performed in a laboratory, lacking real-world variability in patient movement and environmental conditions. Hochhausen et al. [11] extended this work to adults in a Post-Anesthesia Care Unit. Yet, in their work, a manual identification of the region of interest (ROI) was required, making this system less robust. Lorato et al. [12] used a multi-camera setup to monitor respiration in infants in a neonatal intensive care unit. They developed an algorithm with an automatic ROI detection and achieved a mean absolute error (MAE) of 2.07 breaths per minute (BPM) over 152 min.

Lyra et al. [13] applied a deep-learning approach to 26 intensive care unit patients using a YOLOv4-Tiny detector combined with optical flow, reporting an MAE of 2.69 BPM. Even though there is no standardized threshold of MAE to indicate whether a method is suitable to be implemented clinically, it is commonly stated that the MAE for respiration rate should be under 2 BPM [14]. This means that these studies do not meet these requirements yet.

This work represents an important step towards the clinical implementation of thermal cameras for unobtrusive respiration monitoring. We demonstrate and validate a multi-camera thermal imaging setup in adult patients during nocturnal recordings in a clinical setting. In contrast to previous studies that have primarily focused on respiration rate alone, we also assess the extraction and reliability of breath-to-breath measurements, including inter-breath intervals and their variability. In addition, we demonstrated that a single thermal camera could be sufficient for the assessment of respiration in a clinical environment.

## 2. Materials and Methods

### 2.1. Experiment Design

This study, part of the UMOSA (Unobtrusive Monitoring of Obstructive Sleep Apnea) project, was conducted at the Kempenhaeghe Center for Sleep Medicine in Heeze, the Netherlands. The UMOSA study met the ethical principles of the Declaration of Helsinki, the guidelines of Good Clinical Practice, and the current legal requirements. The study was reviewed by the medical ethical committee of the Maxima Medical Center (Veldhoven, the Netherlands, File no: N21.011), and approved by the institutional review board of Kempenhaeghe (File no: CSG_2022_001). All subjects provided written informed consent before participation.

Seven adult patients, aged between 36 and 76 years old, were recruited from patients who were suspected of having sleep-disordered breathing and were scheduled for a clinical polysomnography (PSG) as part of their diagnostic process. Throughout the night, patients stayed in private rooms, where vital signs were monitored using the PSG setup and thermal cameras simultaneously.

### 2.2. Experimental Setup

**Figure 1:**
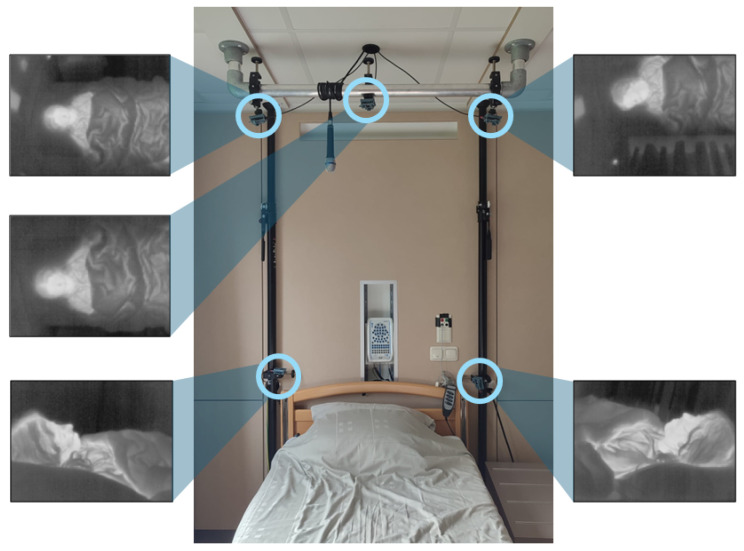
Experimental setup installed in a bedroom at the Kempenhaeghe Centre for Sleep Medicine with the 5 thermal cameras (marked with the circles) and an example frame of each camera simultaneously recorded.

Five thermal cameras were placed symmetrically around the head side of the bed, as shown in Figure 1. Two cameras were placed on the corners of the headboard of the bed, and three cameras were placed (on the right, centre, and left) on a bar 2 m above the bed. The placement of the cameras was based on our previous laboratory study [15]. We limited the use of cameras to positions that would not affect the clinical workflow and therefore no cameras were placed on the sides and foot-end of the bed. Nevertheless, we opted to keep 5 cameras so that we can validate whether a single camera provides similar results when compared to a multi-camera setup.

The cameras used were FLIR Lepton 3.5 (Teledyne FLIR LLC, Wilsonville, OR, USA) connected to the I/O module Pure Thermal 2. These cameras have a resolution of 120 × 160 pixels and an average frame rate of 8.7 Hz. The cameras were divided into two groups, and each group was connected to a computer for the recording of the thermal videos. A third computer was used for the synchronization between the computers and the PSG system.

The PSG system consists of an amplifier docking station placed behind the bed where all the sensors are plugged in. In addition, a microphone is hung on the bar on top of the bed, and a near-infrared (NIR) source and an NIR camera are placed on the ceiling at the foot end of the bed. The sensors that each patient carries are two respiratory belts (on the thorax and on the abdomen), a nasal cannula and a nasal thermistor, and several electrodes are placed on the head, chest, and legs, and an oximeter is placed on one finger.

### 2.3. Data Collection

Recordings were started once the patients were settled in their bedrooms and stopped in the morning. Therefore, the recordings contain moments when the participants are awake and moving, and moments when nurses are interacting with them. The complete dataset consists of approximately 56 h of recordings, where each recording includes 5 videos (one from each camera).

For this study, we specifically focused on data without significant movement or breathing disturbances. Forty-five minutes of data were selected as they provided a representative and sufficient sample. These corresponded to 12 segments from 7 patients, each lasting between 2 and 7 min. These segments were randomly selected by analyzing the reference signals and the videos and manually picking periods with no movement or noise. We ensured that different sleeping orientations of both the head and body were represented in the dataset. Table 1 contains the information on the duration, patient number, and sleeping position of each segment. The corresponding PSG signals were selected. For this analysis, considering that the data selected does not present breathing or movement anomalies, all the reference sensors provide very similar signals. Therefore, the abdominal belt signals were chosen as the reference for this study.

### 2.4. Data Processing

**Figure 2:**
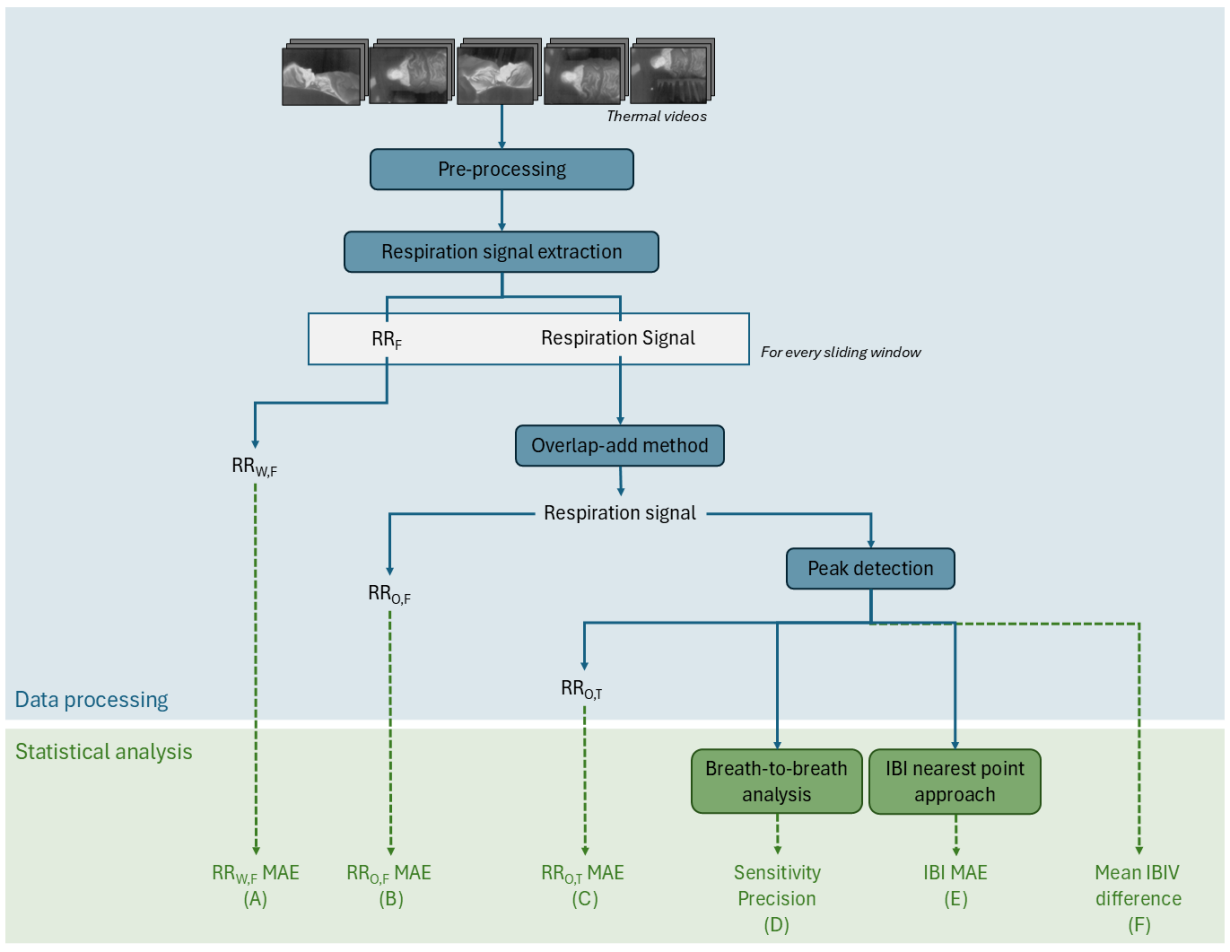
Flowchart with the data processing steps. The blue steps represent the actual data processing performed by the algorithm (top box), while the green steps represent the statistical analysis performed in this paper to evaluate the quality of the algorithm’s outputs (bottom box). IBI—inter-breath interval; IBIV—inter-breath interval variability; F—frequency analysis; MAE—mean absolute error; O—overlap-add method; RR—respiration rate; T—time analysis; W—sliding window method.

The processing steps are summarized in the flowchart of Figure 2. Each thermal video consists of an image sequence of 120 × 160 8-bit pixels. Each pixel value corresponds to a non-absolute temperature. The acquired videos are pre-processed so that the timestamps are constant and the same for each camera. This is done by interpolating the video images to the desired timestamp with an average framerate of 8.7 Hz. The 5 videos are then combined into a 600 × 160 video.

To extract the respiration signal, a pre-existing algorithm developed by Lorato et al. [12] was adapted to our case. The algorithm was designed for respiration monitoring in newborns whose RR lay between 30 and 100 BPM. As for adults, a normal respiration rate (RR) ranges between 12 and 20 BPM, the filtering parameters were set between 5 and 30 BPM, and the sliding window size was changed from 15 s to 45 s [16].

Lorato et al.’s algorithm [12] uses a video input, which can be of one camera or a combination of several cameras, to extract 3 features: the pseudoperiodicity, the clusters of RR, and the thermal gradient. These three features enhance the pixels that are more likely to have respiration information. This is based on the assumption that respiration pixels have a periodic intensity variation, are present in clusters, and, most likely, are located on edges and high-contrast areas. With the resulting product of these features, the highest intensity pixel, is identified. This pixel is the core pixel, and it is considered to be the pixel with the strongest connection to the breathing signal. By thresholding the correlation between the core pixel and the rest of the thermal video pixels, the ROI is defined. This ROI is a group of pixels that are not bound to a specific shape or region and therefore do not rely on the detection of facial features. The average intensity of the ROI is the respiration signal.

This analysis is carried out for each sliding window. For each sliding window, there is a core pixel, an ROI, and a respiration signal. In this step, an RR value can be extracted—the RR_W,F_. This value, obtained for each window, is a result of a frequency analysis. The peak of the frequency spectrum of the signal, which can be seen as the predominant frequency of the signal, is the RR.

Instead of doing an analysis per sliding window, the signals of each window can be combined with the overlap-add method [17]. The final respiration signal corresponds to the whole duration of the segment. In this segment, the same frequency analysis can be done to extract the RR_O,F_.

Another method to compute the RR from a respiration signal is through a time approach. Prior to the implementation of this method, it is required to perform a peak detection. For the peak detection algorithm, it is assumed that the peaks are located on the positive part of the signal since it oscillates around zero, and that the distance between peaks is at least 2 s (assuming an RR lower than 30 BPM). Once the peaks are detected, the temporal distances between the peaks are the inter-breath intervals (IBIs), and the inverse of the average of the IBIs is the RR, which also equals the number of peaks per time unit. When this is carried out for the respiration signal of the whole segment, we obtain the RR_O,T_.

### 2.5. Statistical Analysis

To evaluate the performance of the thermal imaging system, several metrics were computed using the abdominal signal from the PSG system as reference. The green steps in Figure 2 show the various metrics used to evaluate the quality of our thermal camera-based techniques compared to the reference signals. In this section, we will detail how each of these 6 metrics, labeled from (A) to (F), is computed.

Metrics (A), (B), and (C) correspond to the MAE computed between the RR obtained through thermal imaging and the RR from the reference signal. The MAE is calculated through Equation (1), where $x_{i}$ is the measured RR, *x* is the reference RR, and *n* is the number of values compared. The reference RR is computed with the same method as the measured RR. For metric (A) RR_W,F_ MAE, the comparison is done for every sliding window of every segment (*n* = 433) while in metrics (B) RR_O,F_ MAE and (C) RR_O,T_ MAE, only one value per segment is compared (*n* = 12).

(1) $$MAE=\frac{1}{n}\sum_{i=1}^{n}|x_{i}-x|$$

**Figure 3:**
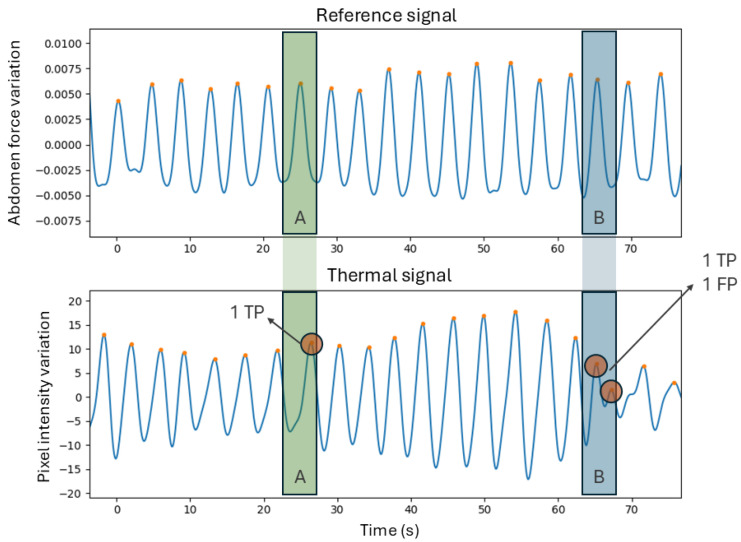
Example of the breath-to-breath analysis method in a fragment of segment 0. Intervals A and B were defined around two peaks in the reference signal, and they were matched to the respective intervals in the thermal signal. Interval A in the thermal signal includes one peak, which will represent a true positive (*TP*), while interval B includes two peaks, which represent one *TP* and one false positive (*FP*).

Metrics (D) are related to the assessment of the breath detection performance, computed through a breath-to-breath comparison method [15]. This method allows for the quantification of the breaths that were correctly identified or missed. This is done by establishing a window that is centered on each peak in the reference signal and limited to half the distance to the neighboring peaks. For each window, if the number of peaks is equal to one, it means that the breath was correctly identified and therefore there is a true positive (*TP*). If the number of peaks equals zero, then the thermal signal did not detect the breath, meaning there is a false negative (*FN*). Finally, if there is more than one peak, then it suggests that there is one *TP* and the rest are false positives (*FPs*). Figure 3 contains a demonstration of this method. For all the segment signals, the *TP*, *FP*, and *FN* were computed to derive sensitivity and precision, whose formulas are present in Equations (2) and (3).

(2) $$Sensivity=\frac{TP}{TP+FN}$$

(3) $$Precision=\frac{TP}{TP+FP}$$

**Figure 4:**
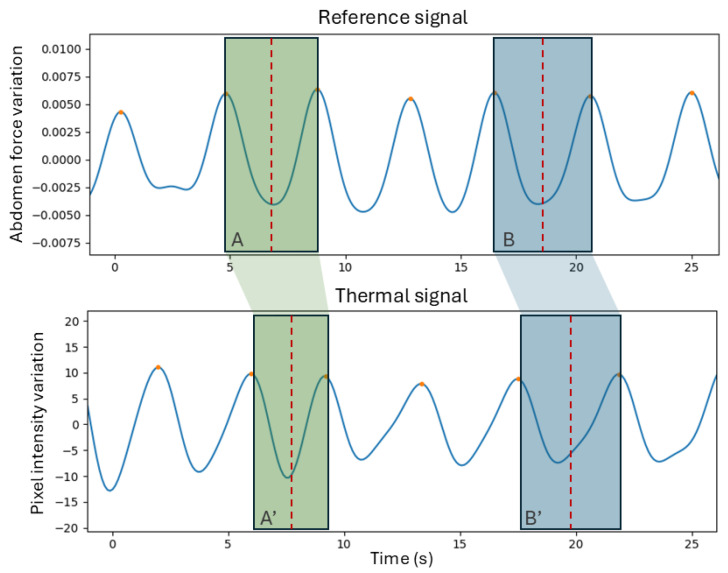
Example of the nearest neighbor method used to match inter-breath intervals (IBIs) in a fragment of segment 0. Intervals A and B were defined in the reference signal, and they were matched to the intervals in the thermal signal whose central point was closest to the central point of the intervals. Interval A was matched to interval A’, and interval B was matched to interval B’.

The IBIs, which correspond to the temporal distance between breaths (or peaks in the signal), are another parameter with clinical relevance and are therefore important to evaluate. This will be represented through metric (E). To compare each individual IBI in the two signals, a method was required to match the IBIs of the reference with the IBIs of the thermal signal. For that, the idea behind the method in [18] was used—a nearest neighbor (NN) approach. This is performed by computing the central points of all IBIs and matching these points in the reference signal to the closest ones (in time) in the thermal signal. This means that every IBI of the reference will be matched, but the opposite is not verified. Missed breaths or extra breaths detected will result in longer or shorter IBIs in the thermal signals that will affect the comparison. Figure 4 contains a graphic representation of this method. A final MAE between the IBIs of all the segments is computed (metric (E)).

Finally, metric F relates to the variability of the IBI signal. This was assessed by computing the ratio of the standard deviation to the mean (SD/mean) for both signals [19,20]. Each signal corresponds to the IBIs over time. The average difference between the IBI variability (IBIV) of the thermal signal and the reference signal is the metric indicator (F).

In addition to doing a statistical analysis to evaluate the performance of this thermal system, a comparison between the five-camera setup and single-camera setup was performed. A previous study showed that a single camera can be enough to accurately monitor respiration [15]; therefore, the complete setup was compared to the setup using the camera at the top central position. This camera is the only camera that would equally capture the face of the patient, independently from the sleeping position. A statistical *t*-test was applied to compare the RR_W,F_ of both configurations [21], and a significance level of 0.05 was used to determine whether differences in performance were statistically significant. This analysis is not present on the diagram of Figure 2.

## 3. Results

**Figure A1:**
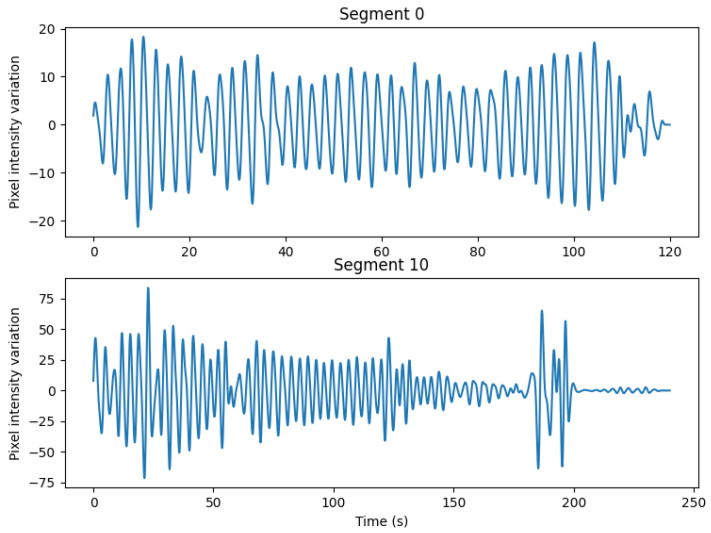
Signal representation for segment 0 and segment 10.

Data acquisition was successfully completed and the 12 segments were isolated, as explained in Section 2.3, and analyzed using the data processing pipeline detailed in Section 2.4. An example of the signals obtained for two representative segments is shown in Figure A1 of Appendix A. In these examples and through visual analysis of the signals, segment 0 is considered a high-quality signal, whereas segment 10 represents the poorest quality signal obtained. The resulting signals were compared to the reference abdominal signal from the PSG system using the approach detailed in Section 2.5. A summary of the overall results is presented in Table 2.

The values of the RR MAE vary between 0.64 and 0.91 BPM depending on the processing method used. The highest one, the RR_W,F_ MAE, was obtained through a frequency analysis for every sliding window. In contrast, the lowest value was the one obtained for the RR_O,T_ MAE, which corresponds to the RR computed using a time domain approach that originates from the detection of peaks on the respiration signal. Through inspection of the results for each segment, it was observed that the segment with the highest MAE was segment 10, the segment that, visually, was considered to have the poorest quality signal.

Breath detection performance was assessed using a breath-to-breath comparison method. In the 12 segments, 633 TPs, 40 FPs, and 24 FNs were quantified. This results in a sensitivity of 96.3%, and a precision of 94.1%, which indicates strong reliability in identifying individual breaths. Table 3 contains the TPs, FPs, and FNs for each segment.

The IBIs, an important clinical parameter, were also evaluated. When matching every single IBI using the NN approach, the system achieved a mean absolute error of 0.48 s. Table 4 contains the IBIV per segment. The IBIV of the reference signal was, on average, 9.2%, while the IBIV of the thermal signal was 11.6%. The average difference between the IBIV was 3.9 percentage points (pp). Segment 10, as mentioned before, was considered to be the noisiest signal and corresponded to the signal with the highest IBIV absolute difference.

Additionally, to validate our previous study [15], the performance of the five-camera setup was compared to a single-camera configuration. This was carried out using a paired *t*-test on the RR_W,F_ values. No statistically significant difference (p>0.05) was found in RR_W,F_ MAE, suggesting that a single well-positioned camera might be sufficient to monitor RR under appropriate clinical conditions.

## 4. Discussion

This study demonstrates the feasibility and effectiveness of using thermal imaging for unobtrusive respiration monitoring in a clinical setting. For this initial validation, we focused on motionless, event-free segments recorded overnight from adult patients, providing an idealized environment to assess the setup and algorithm. While this ensured consistency with earlier studies, extending the evaluation to more challenging conditions, such as periods with movement or artifacts, will be essential to establish the robustness and broader clinical applicability of this technology.

In this work, the thermal imaging system showed strong agreement with the gold-standard polysomnography reference, achieving a mean absolute error between 0.64 and 0.91 BPM in RR estimation. This level of accuracy is comparable to or better than previously reported thermal imaging approaches in controlled laboratory environments, highlighting the performance of the system when applied under clinical conditions [7]. Nevertheless, considering that this study used motion-free segments, further studies are required to validate this system with motion and breathing-disordered periods.

Different methods to compute the RR produced different results. The RR_W,F_ was computed by averaging the frequency-based RR for several sliding windows per segment, while the RR_O,F_ extracted one frequency-based RR on each segment. This means that small RR oscillations, errors, or inaccuracies are not as easily captured with the second method. The RR_O,T_ is a time-based RR value per segment that comes from a prior peak detection algorithm. This algorithm also perfects the results since the peaks are filtered by height and distance. While most of the previous studies done in the field use the RR_W,F_ values, the other two methods are also widely used in clinical practice and, therefore, important to report on and understand their differences.

The breath detection results, with a sensitivity of 96.3%, and a precision of 94.1%, further support the system’s reliability in identifying individual breaths. These values indicate that, overall, the algorithm is both accurate and consistent. Nevertheless, for a clinical application such as sleep monitoring, it is important that the system does not overlook missed breaths that can relate to apneic events. Therefore, the number of FPs and consequently the precision metric should be improved.

Regarding the tracking of inter-breath intervals (IBIs), a mean absolute error of 0.48 s confirms the clinical accuracy of the signal extracted from thermal data. This level of accuracy is essential for assessing sleep-related breathing disorders, such as apnea or hypopnea. Given the selection of motionless and event-free segments, low IBI variability (IBIV) is expected. Moreover, the absolute difference between the IBIV of the thermal signal and that of the reference should be small. A variability difference of 3.9 pp confirms the coherence between the thermal method and the reference signal.

In our previous laboratory work [15], we concluded that one thermal camera could be enough to estimate the respiration rate. Our current results support these findings since there was no statistically significant difference between the five-camera and the best single-camera setup. This is true considering that we used segments without movement and without breathing disorders. In these cases, a simpler system works just as well and is easier to install in a clinical setting. However, in situations with movement or obstructive events like apnea, having multiple cameras or testing different placements of cameras may still be required.

**Figure 5:**
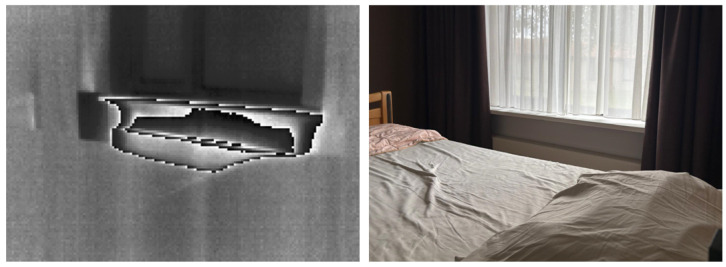
Example of the influence of a heater on a thermal image (**left**) and the corresponding RGB image (**right**). The high-contrast area in the thermal image corresponds to the heater that creates heat waves and high temperature differences with the environment, especially with the window above.

Despite these promising results, several practical challenges were encountered during data acquisition. The presence of heaters in patient rooms introduced thermal noise and fluctuations in the background, which sometimes affected the visibility of the respiratory signal. Figure 5 shows the observed influence of a heater in a thermal image. Additionally, due to the clinical setting, camera positions were occasionally disturbed by patient movement or unintentional contact from healthcare staff, leading to the loss of video signal. These factors highlight the importance of robust camera solutions that can be potentially solved with sturdier mounting structures, a dynamic selection of cameras, and robust video processing algorithms that can isolate the respiration signal even in adverse conditions.

Compared to previous research, this study advances the field by being one of the first to evaluate thermal imaging for respiration monitoring in adult patients within a clinical context, without interfering with patient comfort or the clinical workflow. This supports thermal imaging as a viable, non-contact alternative to traditional sensors, especially in scenarios where sensor placement is challenging.

The analysis in this study was limited to motionless and event-free segments, which, while important for validating the method, do not capture the full variability that is present in typical overnight recordings. Future work should aim to assess the system’s performance during periods of movement or position changes. This might require an algorithm to detect movement and classify those periods as ‘unreliable due to movement’. Additionally, including the assessment of pathological data like obstructive apnea and hypopneas will enrich these findings and the potential of this system.

## 5. Conclusions

This study demonstrates that thermal imaging is a viable, unobtrusive method for monitoring respiration in adult patients during overnight sleep in a clinical environment. The system achieved high accuracy in respiration rate estimation and breath detection, showing strong agreement with the polysomnography reference. Importantly, no significant difference was found between single- and multi-camera setups, confirming the practicality of minimal configurations in clinical settings. Despite environmental and operational challenges, the method proved to be robust and reliable. These findings support the integration of thermal imaging into clinical workflows and highlight its potential as a non-contact alternative for respiratory monitoring. Future work will focus on extending the system’s application to more dynamic sleep conditions and a broader patient population.

## Figures and Tables

**Table 1:** Information on patient number, duration, sleeping position, and head position of the 12 segments selected from the whole dataset.

Segment	Patient	Duration	Sleep Position	Head Orientation
0	1	120 s	Lateral	Left
1	1	180 s	Prone	Right
2	2	180 s	Supine	Right
3	2	240 s	Lateral	Right
4	3	120 s	Lateral	Left
5	3	240 s	Lateral	Right
6	4	180 s	Lateral	Left
7	4	300 s	Lateral	Left
8	5	420 s	Lateral	Left
9	6	180 s	Lateral	Right
10	6	240 s	Supine	Frontal
11	7	300 s	Lateral	Right

**Table 2:** Summary of results for the different statistical analyses.

	Metric	Result
(A)	RR_W,F_ MAE	0.91 BPM
(B)	RR_O,F_ MAE	0.82 BPM
(C)	RR_O,T_ MAE	0.64 BPM
(D)	Sensitivity	96.3%
	Precision	94.1%
(E)	IBI MAE	0.48 s
(F)	Mean IBIV difference	3.9 pp

**Table 3:** Information on the true positives (*TP*), false positives (*FP*), and false negatives (*FN*) detected on each segment.

Segment	*TP*	*FP*	*FN*
0	28	2	1
1	42	3	1
2	42	5	1
3	61	5	2
4	23	1	2
5	45	3	0
6	40	1	1
7	67	2	3
8	102	5	5
9	52	6	0
10	64	2	6
11	67	5	2

**Table 4:** Inter-breath interval variability (IBIV) per segment for the reference signal and the thermal signal, and their absolute difference.

Segment	IBIV Reference (%)	IBIV Thermal (%)	|Δ| IBIV (pp)
0	6.8	13.0	6.3
1	8.2	11.6	3.4
2	15.8	11.8	4.1
3	10.5	14.8	4.3
4	9.7	8.1	1.6
5	13.3	12.8	0.6
6	8.5	8.8	0.3
7	5.6	9.8	4.3
8	11.6	8.8	2.8
9	5.9	10.1	4.2
10	6.7	19.2	12.5
11	7.5	9.9	2.4

## Data Availability

Data sharing is not applicable to this article.

## Abbreviations

The following abbreviations are used in this manuscript:

BPM	Breaths per minute
FN	False negatives
FP	False positives
IBI	Inter-breath interval
IBIV	Inter-breath interval variability
MAE	Mean absolute error
NIR	Near-infrared
NN	Nearest neighbor
pp	Percentage points
PSG	Polysomnography
ROI	Region of interest
RR	Respiration rate
SD	Standard deviation
TP	True positives
